# Higher Intake of Dairy Is Associated with Lower Cardiometabolic Risks and Metabolic Syndrome in Asian Indians

**DOI:** 10.3390/nu14183699

**Published:** 2022-09-07

**Authors:** Ramatu Wuni, Nagarajan Lakshmipriya, Kuzhandaivelu Abirami, Eduard Flores Ventura, Ranjit Mohan Anjana, Vasudevan Sudha, Shanmugam Shobana, Ranjit Unnikrishnan, Kamala Krishnaswamy, Karani Santhanakrishnan Vimaleswaran, Viswanathan Mohan

**Affiliations:** 1 Hugh Sinclair Unit of Human Nutrition, Department of Food and Nutritional Sciences, University of Reading, Reading RG6 6DZ, UK; 2 Department of Food, Nutrition and Dietetics Research, Madras Diabetes Research Foundation, Chennai 600086, India; 3 Dr. Mohan’s Diabetes Specialties Centre, IDF Centre of Excellence in Diabetes Care, Chennai 600086, India; 4 The Institute for Food, Nutrition and Health (IFNH), University of Reading, Reading RG6 6AP, UK

**Keywords:** metabolic syndrome, Asian Indians, dairy intake, fermented dairy, unfermented dairy, CURES

## Abstract

There is conflicting evidence about the association between dairy products and cardiometabolic risk (CMR). We aimed to assess the association of total dairy intake with CMR factors and to investigate the association of unfermented and fermented dairy intake with CMR in Asian Indians who are known to have greater susceptibility to type 2 diabetes and cardiovascular diseases compared to white Europeans. The study comprised 1033 Asian Indian adults with normal glucose tolerance chosen from the Chennai Urban Rural Epidemiological Study (CURES). Dietary intake was assessed using a validated open-ended semi-quantitative food frequency questionnaire. Metabolic syndrome (MS) was diagnosed based on the new harmonising criteria using central obesity, dyslipidaemia [low high-density lipoprotein cholesterol (HDL) and increased serum triglycerides (TG)], hypertension and glucose intolerance. Increased consumption of dairy (≥5 cups per day of total, ≥4 cups per day of unfermented or ≥2 cups per day of fermented dairy) was associated with a lower risk of high fasting plasma glucose (FPG) [hazards ratio (HR), 95% confidence interval (CI): 0.68, 0.48–0.96 for total dairy; 0.57, 0.34–0.94 for unfermented dairy; and 0.64, 0.46–0.90 for fermented dairy; *p* < 0.05 for all] compared to a low dairy intake (≤1.4 cups per day of total dairy; ≤1 cup per day of unfermented dairy; and ≤0.1 cup per day of fermented dairy). A total dairy intake of ≥5 cups per day was also protective against high blood pressure (BP) (HR: 0.65, 95% CI: 0.43–0.99, *p* < 0.05), low HDL (HR: 0.63, 95% CI: 0.43–0.92, *p* < 0.05) and MS (HR: 0.71, 95% CI: 0.51–0.98, *p* < 0.05) compared to an intake of ≤1.4 cups per day. A high unfermented dairy intake (≥4 cups per day) was also associated with a lower risk of high body mass index (BMI) (HR: 0.52, 95% CI: 0.31–0.88, *p* < 0.05) compared to a low intake (≤1 cup per day), while a reduced risk of MS was observed with a fermented dairy intake of ≥2 cups per day (HR: 0.71, 95% CI: 0.51–0.98, *p* < 0.05) compared to an intake of ≤0.1 cup per day. In summary, increased consumption of dairy was associated with a lower risk of MS and components of CMR.

## 1. Introduction

Asian Indians have been shown to have distinct biochemical and clinical characteristics that put them at risk of type 2 diabetes (T2D) and cardiovascular diseases (CVDs) [1,2,3,4]. The distinct features include central obesity, dyslipidaemia, insulin resistance, increased levels of visceral fat, total fat, and propensity to beta cell dysfunction [1,2,3,4]. The components of the ‘Asian Indian Phenotype’ are included in the metabolic syndrome (MS), which refers to a group of interconnected risk factors that make an individual susceptible to CVDs and T2D [5]. According to a systematic review and meta-analysis involving 133,926 participants from 111 studies [6], MS affects 1 in 3 adults in India, and the prevalence is higher among people in urban areas (32%) than those living in rural areas (22%). MS is associated with increased CVDs and all-cause mortality [7,8], warranting studies in Asian Indians who are known to have a predisposition to MS.

The existence of an entity called MS is surrounded by controversies, partly due to variations in the definition of MS [9,10,11,12]. However, it is generally agreed that the risk factors of central obesity, high blood pressure (BP), elevated levels of triglycerides (TG), low concentration of high-density lipoprotein cholesterol (HDL) and elevated fasting plasma glucose (FPG) tend to co-exist and are important indicators of an individual’s risk of CVDs and T2D [9,10,11,12,13]. The increasing prevalence of these risk factors has been linked to genetic and environmental factors [1,14,15,16,17], and there is growing interest in the role of different types of food in the development of MS [1,5,16,17]. Several studies have reported a protective effect of dairy consumption on the risk of MS [5,18,19,20,21]. Consumption of at least two servings of dairy per day compared to no dairy intake, has been linked to a lower prevalence of MS [5]. Increased consumption of dairy (>7 times per week) was also found to be associated with a reduced risk of MS and central obesity compared to no dairy intake [21]. However, one study [22] reported that participants who did not consume milk had a lower risk of insulin resistance and MS compared to those who drank milk, making the findings inconsistent. Moreover, it has been suggested that fermented dairy might confer greater anti-inflammatory and cardiometabolic benefits than unfermented dairy [23,24]. Possible mechanisms for the proposed benefits of fermented dairy include the action of microbial cultures on gut microbiota, changes in lipid and glyceride profiles and the release of more bioactive compounds involved in regulating several metabolic and immune pathway genes [23,24,25].

Furthermore, consumption of dairy is high among Asian Indians [26,27,28] who also have a high prevalence of MS [6,29,30]. An examination of the dietary profile of 2042 Asian Indian participants [26] showed that, dairy intake was within the national recommendation of 300 g/day (g/day) [31]. However, despite dairy consumption being linked to lower risk of MS [5,18,19,20], few studies have examined the impact of dairy intake on the risk of MS in Asian Indians. Hence, the present study sought to investigate the association of total dairy consumption with MS and components of cardiometabolic risk (CMR) in Asian Indians. We also aimed to determine the association of fermented and unfermented dairy products with MS and components of CMR.

## 2. Methods

### 2.1. Study Population

The current study consisted of 1033 adults with normal glucose tolerance chosen from the Chennai Urban Rural Epidemiological Study (CURES), and details of the study design have been given in previous publications [1,15,32,33,34]. In brief, a total of 26,001 adults were recruited between 2001 to 2003 from the urban part of Chennai in Southern India through systematic random sampling, and the follow-up study was conducted between 2012 and 2013 and consisted of 2410 participants. The sample for the current study was chosen from the follow-up cohort as shown in Supplementary Figure S1. Approval was obtained from the Institutional Ethics Committee, and written informed consent obtained from all the study participants.

### 2.2. Data Collection

Demographic (including medical history and physical activity), anthropometric, biochemical and dietary data were collected both at baseline (2001–2003) and after 10 years (2012–2013) using a structured, pretested, and validated interviewer-administered questionnaire [35]. Family history of diabetes was considered as positive if either parents or sibling/s had diabetes. Smokers were defined as those who were currently smoking, and alcohol use was defined as current alcohol consumption.

Height, weight, waist circumference (WC) and BP were measured using standardised techniques [32], and body-mass index (BMI) was calculated as weight in kilograms (kg) divided by height in meters squared (m^2^). Biochemical analyses, including fasting plasma glucose (FPG) and lipids, were performed in all individuals; in addition, plasma glucose estimation 2 h after a 75 g oral glucose load was performed in individuals without diabetes [32]. Biochemical analyses were performed in a laboratory certified by the National Accreditation Board for Testing and Calibration Laboratories and the College of American Pathologists on a Hitachi 912 autoanalyzer (Hitachi, Mannheim, Germany) using kits supplied by Roche Diagnostics (Basel, Switzerland) for estimation of plasma glucose (GOD-POD method).

#### 2.2.1. Outcome Ascertainment

##### General Obesity

General obesity was defined as BMI ≥ 25 kg/m^2^ and overweight as BMI ≥ 22.9 kg/m^2^ in accordance with the Asia Pacific guidelines [36].

##### Metabolic Syndrome

MS was diagnosed based on the new harmonising criteria [37]. Individuals with any three of the following abnormalities viz. high WC (Asia Pacific cut-off ≥80 cm for female, ≥90 cm for male), hypertriglyceridemia [serum TG ≥ 1.70 mmol/L (≥150 mg/dL)], low HDL [male participants ≤ 1.04 mmol/L (≤40 mg/dL); female participants ≤ 1.30 mmol/L (≤50 mg/dL)], abnormal glucose metabolism [defined as FPG ≥ 5.6 mmol/L (≥100 mg/dL)] and elevated BP [systolic BP (SBP) ≥ 130 mmHg or diastolic BP (DBP) ≥ 85 mmHg] were considered to have MS.

The term “cardiometabolic risk” was first employed by the American Diabetes Association as an umbrella term to include all the risk factors for diabetes and CVD [38]. The components of CMR given in the present analysis are central and general obesity; elevated levels of triglycerides, total cholesterol and LDL and reduced HDL concentration; hyperglycaemia; hypertension; and insulin resistance.

#### 2.2.2. Dietary Assessment

Dietary intake was assessed by trained dietitians using a validated open-ended semi-quantitative 222-item food frequency questionnaire (FFQ) both at baseline and follow-up. The FFQ was designed to estimate the usual dietary intake of participants, the development and validation of which have been described elsewhere [35]. The FFQ included both the frequency as well as the servings of food items consumed by the individuals which was then converted to standardised portion sizes. However, any new food item reported (new market foods over 10-year period) during the follow-up period was updated in the in-house Nutritional Epidemiology (‘EpiNu’) software. Dairy intake was estimated from the FFQ using the ‘EpiNu’ software. Total dairy intake consists of unfermented plain milk and milk included in tea and coffee; Indian milk sweets and desserts; and fermented milk, which consists of Indian yoghurt (curd) and buttermilk. The ‘EpiNu’ software which contains information on the nutritional composition of food that is mainly consumed in the Chennai area was developed for the local population using recipes from a wide range of sources, including fast-food and home-made. Details of the development of the ‘EpiNu’ software are available in a previous publication [35].

### 2.3. Statistical Analyses

Statistical analyses were performed using SAS software version 9.4 (SAS Institute Inc., Cary, NC, USA). All food groups and nutrients were energy adjusted by the residual method [39]. As nutrients and food groups were not normally distributed, estimates were expressed in median and interquartile range (IQR). The Mann-Kruskal Wallis test was used to compare differences between the medians of continuous variables, and the chi-squared test was used to test differences in proportions. The lowest, medium and highest intakes of total dairy, unfermented and fermented dairy were derived by stratifying the data into deciles and regrouping as lowest (quartile 1(Q1)–quartile 4 (Q4)), medium (Q5–Q8) and highest intake (Q9–Q10) to test the association with CMR using the regression model. The hazard ratio (HR) for incidence of CMR and MS in each group of dairy intake (lowest intake, medium intake and highest intake) and its subdivision (fermented and unfermented) was calculated using Cox proportional hazards analysis. Potential confounders were identified by the univariate analysis and entered simultaneously into the multiple Poisson regression model with *p*-value < 0.2. The model was adjusted for age, sex, BMI, income, smoking, alcohol, major cooking oil, total poly unsaturated fatty acids (PUFA) (g), added sugar (g), physical activity level (PAL), total energy (kcal) and tea and coffee intake. The linear trend across the lowest, medium and highest dairy intake and incidence of CMR and MS were tested with the regression model [40]. Difference between the dairy product and its subdivisions was assessed using the Kruskal-Wallis test for all the continuous variables. The *p* values were tested for statistical significance at <0.05 level.

## 3. Results

### 3.1. Characteristics of the Study Participants

**Figure 1:**
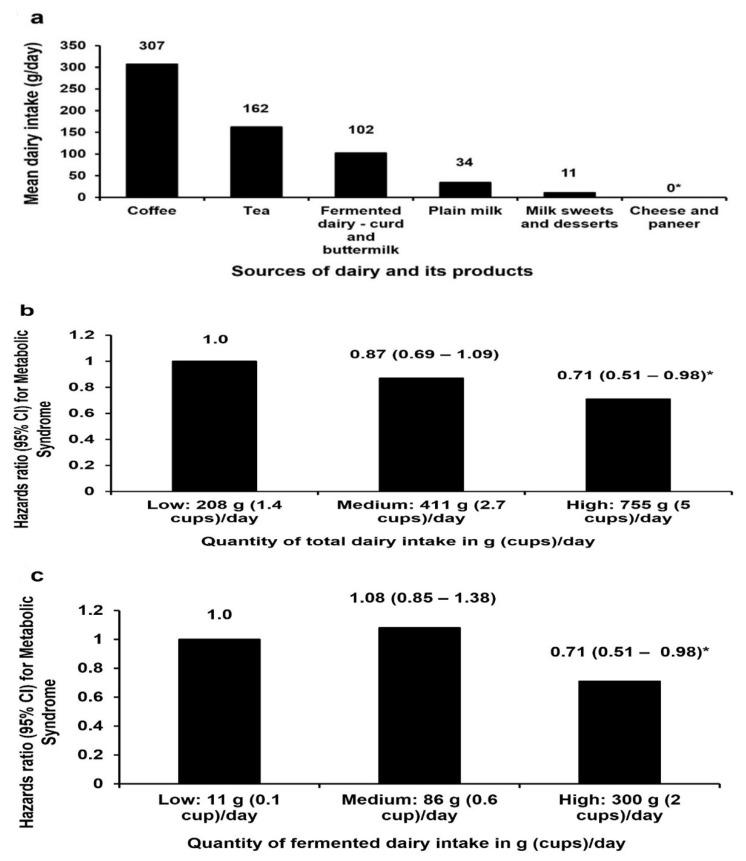
(**a**) **The sources of dairy and its products among the Chennai urban adults**. Milk sweets and desserts include Indian milk sweets, ice cream, milk shakes and other milk beverages. * Cheese and paneer intake was reported by only three individuals in the sample, and this resulted in a median value of 0. (**b**) **Total dairy consumption and its association with metabolic syndrome**. Data presented as median. * *p*-value < 0.05 considered as significant. Adjusted variables are age (years), sex, BMI, income, smoking, alcohol, major cooking oil, total poly unsaturated fatty acids (PUFA) (g), added sugar (g), total energy (kcal) and tea and coffee intake. (**c**) **Fermented dairy consumption and its association with metabolic syndrome.** Data presented as median. * *p*-value < 0.05 considered as significant. Adjusted variables are age (years), sex, BMI, income, smoking, alcohol, major cooking oil, PUFA (g), added sugar (g), physical activity level, total energy (kcal) and tea and coffee intake.

The median age of the study participants was 36 (IQR: 15) years. As shown in Table 1, smoking and alcohol consumption were reported by 16% and 23% of participants, respectively. Nearly half of the participants (44%) had a family history of diabetes. The median SBP (113 mmHg), DBP (72 mmHg), FPG (84 mg/dL) and postprandial glucose (106 mg/dL) were within the normal ranges. Consumption of tea and coffee was the main source of dairy (80%) as shown in Figure 1a and Table 2. The medians of the lowest, medium, and the highest total dairy intake were 208, 411 and 755 g/day (1.4, 3 and 5 cups per day), respectively.

### 3.2. Association of Total Dairy Consumption and Components of CMR

A total dairy intake of ≥5 cups compared to ≤1.4 cups per day was associated with a decreased risk of three of the components of CMR [high BP, FPG and low HDL] and MS as shown in Table 3 and Figure 1b, respectively. A decreased incidence of two of the components of CMR (high FPG and low HDL) was also observed among individuals in the medium total dairy intake group (≥3 cups per day) compared to those in the low total dairy intake group (≤1.4 cups per day) (Table 3). There was no association between total dairy intake and insulin resistance as shown in Supplementary Figure S2.

**Table 3:** Total Dairy Consumption and its Association with Components of Cardiometabolic Risk.

	Hazards Ratio (95% Confidence Interval)		
	*Lowest Intake* Q1–Q4	*Medium Intake* Q5–Q8	*Highest Intake* Q9–Q10
*Total Dairy Products (g/Day)*	*208* (*116*) 1.4 Cups	*411* (*144*) 3 Cups	*755* (*228*) 5 Cups
Blood pressure (mmHg) ≥ 140/90	1 (ref)	0.82 (0.63–1.08)	0.65 (0.43–0.99) *
BMI (kg/m^2^) ≥ 22.9	1 (ref)	0.84 (0.66–1.08)	0.78 (0.53–1.15)
Waist circumference (cm) (>80: F; >90: M)	1 (ref)	0.87 (0.7–1.09)	0.87 (0.62–1.24)
Total cholesterol (>200 mg/dL)	1 (ref)	0.72 (0.51–1.01)	0.70 (0.42–1.18)
Triglyceride (>150 mg/dL)	1 (ref)	1.05 (0.76–1.44)	0.74 (0.45–1.22)
High-density lipoprotein (mg/dL) (≤40: F; ≤50: M)	1 (ref)	0.74 (0.59–0.93) *	0.63 (0.43–0.92) *
Low-density lipoprotein (>100 mg/dL)	1 (ref)	0.95 (0.77–1.17)	0.83 (0.61–1.12)
Fasting plasma glucose (>100 mg/dL)	1 (ref)	0.75 (0.6–0.95) *	0.68 (0.48–0.96) *

Data presented as median (interquartile range). * *p*-value < 0.05 considered as significant. Adjusted variables are age, sex, BMI, income, smoking, alcohol, major cooking oil, total poly unsaturated fatty acids (PUFA) (g), added sugar (g), physical activity level, total energy (kcal) and tea and coffee intake (g/day).

### 3.3. Association of Unfermented Dairy Consumption and Components of CMR

Consumption of 4 cups per day or more of unfermented dairy was associated with a lower incidence of high BMI and FPG (Table 4) compared to an intake of ≤1 cup per day of unfermented dairy. There was no significant association between unfermented dairy intake and MS (Supplementary Figure S2).

### 3.4. Association of Fermented Dairy Consumption and Components of CMR

Consumption of 2 cups per day or more of fermented dairy was associated with a lower incidence of high FPG (Table 4) compared to an intake of ≤0.1 cups per day. A high fermented dairy intake (≥2 cups per day) was also associated with a lower risk of MS compared to a low fermented dairy intake (≤0.1 cups per day) (hazards ratio (HR): 0.71, 95% confidence interval (CI): 0.51–0.98, *p* < 0.05) as shown in Figure 1c.

**Table 4:** Fermented and Unfermented Dairy Consumption and its Association with Components of Cardiometabolic Risk.

	Hazards Ratio (95% Confidence Interval)					
	Unfermented Dairy Products (g/Day)			Fermented Dairy Products (g/Day)		
	*Lowest Intake* Q1–Q4	*Medium Intake* Q5–Q8	*Highest Intake* Q9–Q10	*Lowest Intake* Q1–Q4	*Medium Intake* Q5–Q8	*Highest Intake* Q9–Q10
*Dairy Product* (*g/Day*)	*138* (*86*) 1 Cup	*290* (*103*) 2 Cups	*581* (*175*) 4 Cups	*11* (*23*) 0.1 Cup	*86* (*54*) 0.6 Cup	*300* (*116*) 2 Cups
Blood pressure (mmHg) ≥ 140/90	1 (ref)	1.01 (0.73–1.41)	0.75 (0.45–1.27)	1 (ref)	0.83 (0.63–1.10)	0.71 (0.49–1.03)
BMI (kg/m^2^) ≥ 22.9	1 (ref)	0.70 (0.50–0.99)	0.52 (0.31–0.88) *	1 (ref)	0.83 (0.63–1.10)	0.71 (0.49–1.03)
WC (cm) (>80: F; >90: M)	1 (ref)	0.91 (0.71–1.15)	0.89 (0.62–1.26)	1 (ref)	1.12 (0.92–1.37)	1.03 (0.81–1.34)
Total cholesterol (>200 mg/dL)	1 (ref)	0.78 (0.5–1.22)	0.59 (0.3–1.16)	1 (ref)	10 (0.72–1.39)	0.83 (0.54–1.28)
Triglyceride (>150 mg/dL)	1 (ref)	0.83 (0.57–1.2)	0.68 (0.38–1.22)	1 (ref)	1.14 (0.84–1.53)	0.98 (0.69–1.4)
HDL (mg/dL) (≤40: F; ≤50: M)	1 (ref)	1.02 (0.77–1.34)	0.93 (0.63–1.37)	1 (ref)	0.86 (0.69–1.06)	0.76 (0.57–1.01)
LDL (>100 mg/dL)	1 (ref)	0.92 (0.71–1.19)	0.77 (0.53–1.13)	1 (ref)	1.09 (0.9–1.33)	0.88 (0.69–1.13)
Fasting plasma glucose (>100 mg/dL)	1 (ref)	0.62 (0.44–0.88)	0.57 (0.34–0.94) *	1 (ref)	0.96 (0.74–1.24)	0.64 (0.46–0.90) *

Data presented as median (interquartile range). * *p*-value < 0.05 considered as significant. Adjusted variables are age, sex, BMI, income, smoking, alcohol, major cooking oil, total polyunsaturated fatty acids (PUFA) (g), added sugar (g), physical activity level (PAL), total energy (kcal) and tea and coffee intake. HDL—high-density lipoprotein cholesterol; LDL—low-density lipoprotein cholesterol; BMI—body mass index; WC—waist circumference.

## 4. Discussion

The present study has found evidence of a protective effect of dairy consumption against CMR factors in Asian Indians. We found a reduced risk with an increased intake of dairy products, where consumption of ≥5 cups per day of total, ≥4 cups per day of unfermented or ≥2 cups per day of fermented dairy was associated with a reduced risk of high FPG. A total dairy intake of ≥5 cups per was also associated with a lower risk of high BP, low HDL and MS. Consumption of ≥4 cups per day of unfermented dairy was also associated with a decreased incidence of high BMI; while an intake of ≥2 cups per day of fermented dairy was also associated with a lower risk of MS. Given that Asian Indians have high prevalence of CVDs and T2D [1,2,3,26], these findings are of public health importance. India is the largest producer of milk and it is commonly consumed by all classes of income groups, providing value for money and nutrients [27]. The results indicate that increasing the consumption of dairy products might help to reduce the risk of MS and its individual components in Asian Indians.

At baseline, the most widely consumed dairy products were reported to be tea and coffee with milk [26], and the same trend continued in the follow-up period after 10 years. In the Chennai area, a large quantity of milk is typically used in the preparation of tea and coffee, hence milk added to tea and coffee is a main source of dairy in the study population. Given that tea and coffee intake may independently influence the risk of CVDs (Supplementary Table S1), we adjusted for tea and coffee intake in our analysis. Our findings are consistent with previous studies in which dairy consumption showed a protective effect against MS [5,18,19,20,21]. In the Prospective Urban Rural Epidemiology (PURE) study [5], a large, multinational cohort study involving 112,922 individuals from 21 countries with a median follow-up of 9.1 years, a higher total dairy intake (≥2 servings per day) compared with no intake, was associated with a decreased prevalence of MS [odds ratio (OR), 0.76; 95% CI, 0.71–0.80; *p_trend_* < 0.0001]. Similarly, the Brazilian Longitudinal Study of Adult Health (ELSA-Brasil), which involved 9835 participants [18], observed that total dairy intake was inversely associated with metabolic risk score (Beta = −0.04 ± 0.01, *p* = 0.009). The French Data from the Epidemiological Study on the Insulin Resistance Syndrome (DESIR) [20], a cohort study of 3435 participants also observed a negative association between consumption of dairy products, except cheese, and incidence of MS (OR, 0.88; 95% CI, 0.79–0.97; *p* = 0.01) and impaired fasting glycaemia/T2D (OR, 0.85; 95% CI 0.76–0.94; *p* = 0.001). A prospective study of 7240 Koreans [21] also reported that, a high consumption of dairy (≥7 times a week) was associated with a decreased risk of MS (HR, 0.72; 95% CI, 0.62–0.84; *p_trend_* < 0.001) compared to no consumption of dairy. Overall, these findings indicate that consumption of dairy might be beneficial in reducing the risk of MS in different ethnic groups, but large dietary intervention studies will help to corroborate the findings.

The inverse association between dairy consumption and the risk of individual components of CMR observed in our study is also consistent with previous studies. In the PURE study [5], a higher total dairy intake (≥2 servings per day) compared to no intake, was associated with a decreased incidence of hypertension (HR, 0.89; 95% CI, 0.82–0.97; *p_trend_* = 0.02) and T2D (HR 0.88; 95% CI, 0.76–1.02, *p_trend_* = 0.01). The Caerphilly Prospective Study of 2512 men [41] also reported that participants in the highest milk consumption group had a 10.4 mmHg lower SBP (*p_trend_* = 0.023) than those who did not consume milk after a 22.8 year follow-up. This study [41] also observed lower levels of glucose (*p_trend_* = 0.032) with increasing intake of milk and dairy products. Furthermore, a cross-sectional study of 205 Indian participants with MS [42] showed that, consumption of milk and milk products (>4 servings/day) was associated with a lower risk of hypertension (OR, 0.54 95% CI, 0.18–1.67). A study involving 133 Indian women with gestational diabetes [43] also found an inverse association between consumption of dairy products and adverse neonatal outcomes (OR, 0.14, 95% CI, 0.02–0.8; *p* = 0.03). Moreover, a systematic review of randomised controlled trials [44] reported that dairy intake had a beneficial effect on body weight. All in all, the findings call for large, randomised trials to confirm the effect of dairy products on BP, BMI and blood glucose levels.

Our finding of a positive association between dairy intake and high HDL is also supported by a cohort study of 11,377 Norwegian participants (The Tromsø Study) [45] where consumption of cheese was positively associated with HDL concentration (Beta = 0.02 mmol/L, 95% CI, 0.01–0.03)). However, this association was only observed for total dairy intake in our study. The study [45] also reported that, a high intake of fermented dairy (250 g/day) was associated with lower TG concentration (Beta = −1.11, 95% CI, −1.96 to −0.24; *p* = 0.01) than a low intake, but this was not observed in our study. One possible explanation is that, cheese was a main part of fermented dairy in the Norwegian study [45] while in our study, the median intake of cheese was zero. On the whole, the findings indicate a need for large scale randomised trials to confirm the association of dairy products with blood lipids.

The average intake of SFA (% of energy) for this study population, Chennai urban area was 9% of total energy intake (TEI), which is within the recommended daily allowance of <10% of TEI [46]. Dairy is known to contain high amounts of SFA which is linked to elevated LDL concentration and high risk of CVDs leading to concerns about the health benefits of dairy, with some people resorting to low-fat dairy alternatives [17,47]. However, it has been noted that, SFAs are a large group of fatty acids, and their effects may vary depending on the type of food [17]. Moreover, a large multinational cohort study of 136,384 individuals from 21 countries (PURE) [17] observed no significant association between higher intake of SFA from dairy sources and total mortality or major CVD. Furthermore, odd chain fatty acids are the major SFAs in milk and they have been associated with better CVD outcomes with regards to lipids [48,49]. The association of dairy intake with favourable lipid levels has also been linked to the presence of oleic acid, a monounsaturated fatty acid (MUFA) in dairy products [45] which is known to increase the concentration of HDL and lower the levels of LDL and TG [50,51,52]. Fatty acids derived from milk have also been associated with a decrease in the number of small dense LDL particles, which is linked to a favourable lipid profile since small dense LDL is negatively associated with HDL and positively associated with TG and fasting insulin levels [53]. Milk is a rich source of different nutrients [17,47], and it has been suggested that the protective effect of dairy consumption on the risk of MS is dependent on the individual as well as joint effect of the different nutrients [54,55]. Milk protein is believed to suppress angiotensin I-converting enzyme, which is involved in BP regulation [56]. Milk is also a rich source of potassium, which helps in regulating BP [57]. Whey protein derived from milk has also been reported to influence glucose levels through its involvement in the regulation of gastrointestinal hormones [55]. Fermented dairy is believed to confer greater anti-inflammatory and cardiometabolic benefits than unfermented dairy [23,24], but intake of fermented dairy was relatively low in this study, and this could have influenced our findings of fewer associations between fermented dairy and CMR. It has also been suggested that, the associations of dairy with blood lipids may be impacted by dairy matrix and fat content [45]. Moreover, findings from a large mendelian randomisation analysis of 1,904,220 individuals from three population-based studies [58] indicate that, genetic variants linked to milk consumption, might also influence BMI and lipid levels, suggesting that multiple factors are involved in the association of dairy intake with reduced risk of MS.

The strength of our study is the large sample size and the use of validated instruments in a well-characterised population. This study is one of few studies which have examined the association of total, unfermented and fermented dairy with the risk of MS in Asian Indians. Our study has some limitations. Comparing the benefits of fermented and unfermented dairy intake was not possible due to the relatively low intake of fermented dairy compared to unfermented dairy. Additionally, we did not investigate the effect of individual dairy products on the risk of MS. Furthermore, the fat content of the dairy products was not analysed in our study. Coffee and tea might also influence CVD risk independently as shown in Supplementary Table S1, but data on intake of caffeine and phenolic compounds was not available. However, we adjusted for coffee and tea intake in the regression model. Moreover, evidence from nutrigenetic studies shows that genetic variants might be involved in modifying responses to diet, which is outside the scope of this study. Nonetheless, our findings support previous work and add to the evidence linking dairy consumption to lower risk of MS and components of CMR.

## 5. Conclusions

We found that increased consumption of dairy (≥5 cups per day of total, ≥4 cups per day of unfermented or ≥2 cups per day of fermented dairy) was associated with a lower risk of high FPG. A total dairy intake of ≥5 cups per day was also protective against high BP, low HDL and MS. A high unfermented dairy intake (≥4 cups per day) was also associated with a lower risk of high BMI, while a reduced risk of MS was observed with a fermented dairy intake of ≥2 cups per day. The findings indicate that increasing the consumption of dairy might help to reduce CMR factors (high BP, BMI, FPG and low HDL) and MS in Asian Indians. Larger studies are needed to confirm our findings. Once our findings are confirmed, dietary guidelines focusing on increasing the consumption of dairy might be effective in reducing the risk of MS and components of CMR in Asian Indians.

## Figures and Tables

**Table 1:** Baseline Characteristics of the Study Population (*n* = 1033).

Variables	Overall Median (Interquartile Range)/ *n* (%)
Age (years)	36 (15)
*Gender n (%)*	
Men *n* (%)	433 (42)
Women *n* (%)	600 (58)
Smoking (yes) *n* (%)	160 (15)
Alcohol (yes) *n* (%)	242 (23)
*Income per month n (%)*	
INR. < 2000	24 (2)
INR. 2000–5000	197 (19)
INR. 5000–10,000	415 (40)
INR. > 10,000	397 (39)
Family history of diabetes (yes) *n* (%)	449 (43)
Weight (kg)	58 (17)
BMI (kg/m^2^)	23.2 (6.2)
Waist circumference (cm)	84 (16)
Systolic BP (mmHg)	113 (19)
Diastolic BP (mmHg)	72 (13)
Fasting blood glucose (mg/dL)	84 (12)
Postprandial blood glucose (mg/dL)	106 (33)
Total Cholesterol (mg/dL)	175 (47)
Triglyceride (mg/dL)	96 (65)
High density lipoprotein (mg/dL)	42 (13)
Low density lipoprotein (mg/dL)	109 (39)

Data presented as median (interquartile range) for continuous variables; and as number (*n*) (%) for categorical variables. INR—Indian rupees; BMI—body mass index; BP—blood pressure.

**Table 2:** Consumption of Dairy and its Products (g/day).

	Median (Interquartile Range)		
Dairy and Its Products (g/Day)	*Lowest Intake* Q1–Q4	*Medium Intake* Q5–Q8	*Highest Intake* Q9–Q10
Total dairy products	208 (116)	411 (144)	755 (228)
Fermented dairy products (curd and buttermilk)	32 (66)	75 (119)	167 (215)
Milk	10 (39)	37 (94)	74 (148)
Tea and coffee (contribution by milk)	118 (118)	235 (176)	471 (353)
Milk sweets and desserts (milk sweets, ice cream, milk shake and other milk beverages)	2 (8)	3 (10)	5 (22)

## Data Availability

The data presented in this study are available on request from the corresponding author. The data are not publicly available due to [ethical reasons].

## Supplementary Materials

The following supporting information can be downloaded at: https://www.mdpi.com/article/10.3390/nu14183699/s1, Figure S1: Study Design; Table S1: Tea and coffee consumption and its association with components of cardiometabolic risk; Figure S2: (a) total dairy intake and insulin resistance, (b) unfermented dairy intake and metabolic syndrome.
