# Supplementary material for: Higher Intake of Dairy Is Associated with Lower Cardiometabolic Risks and Metabolic Syndrome in Asian Indians
Source: Nutrients. 2022 Sep 7;14(18):3699. doi: 10.3390/nu14183699 (PMC9503034; doi:10.3390/nu14183699)
Supplement: Supplementary file 1 [file nutrients-14-03699-s001.zip › nutrients-1842428-supplementary.pdf]

Supplementary Table S1. Tea and coffee consumption and its Association with Components of Cardiometabolic Risk

	**Hazards Ratio (95% Confidence Interval)**		
	Lowest intake	***Medium intake***	***Highest intake***
Tea and coffee (g/day)	170 (219)	470 (144)	1176(470)
Milk (g/day) in the tea/coffee	***85***	***235***	***588***
Serving in cup	3/4 cups	2.5 cups	5 cups
Blood pressure (mmHg) ≥ 140/90	1 (ref)	0.84(0.65 – 1.07)	0.60(0.39 – 0.91)*
BMI (kg/m^2) ≥ 22.9	1 (ref)	0.78(0.59 – 1.03)	0.56(0.36 – 0.88)*
Waist circumference (cm) (> 80: F; > 90: M)	1 (ref)	0.91(0.76 – 1.09)	0.81(0.62 – 1.07)
Total cholesterol (> 200 mg/dL)	1 (ref)	0.63(0.44 – 0.90)	0.57(0.34 – 0.95)*
Triglyceride (> 150 mg/dL)	1 (ref)	0.73(0.54 – 0.99)*	0.60(0.38 – 0.93)*
High density lipoprotein (mg/dL) (≤ 40: F; ≤ 50: M)	1 (ref)	0.86(0.70 – 1.06)	0.77(0.55 – 1.08)
Low density lipoprotein (> 100 mg/dL)	1 (ref)	0.80(0.66 – 0.97)*	0.77(0.58 – 1.03)
Fasting plasma glucose (> 100 mg/dL)	1 (ref)	0.90(0.69 – 1.17)	1.02(0.69 – 1.50)

Data presented as median (interquartile range). **P*-value < 0.05 considered as significant. Adjusted variables are age, sex, BMI, income, smoking, alcohol, major cooking oil, total poly unsaturated fatty acids (PUFA) (g), added sugar (g), physical activity level, and total energy (kcal).

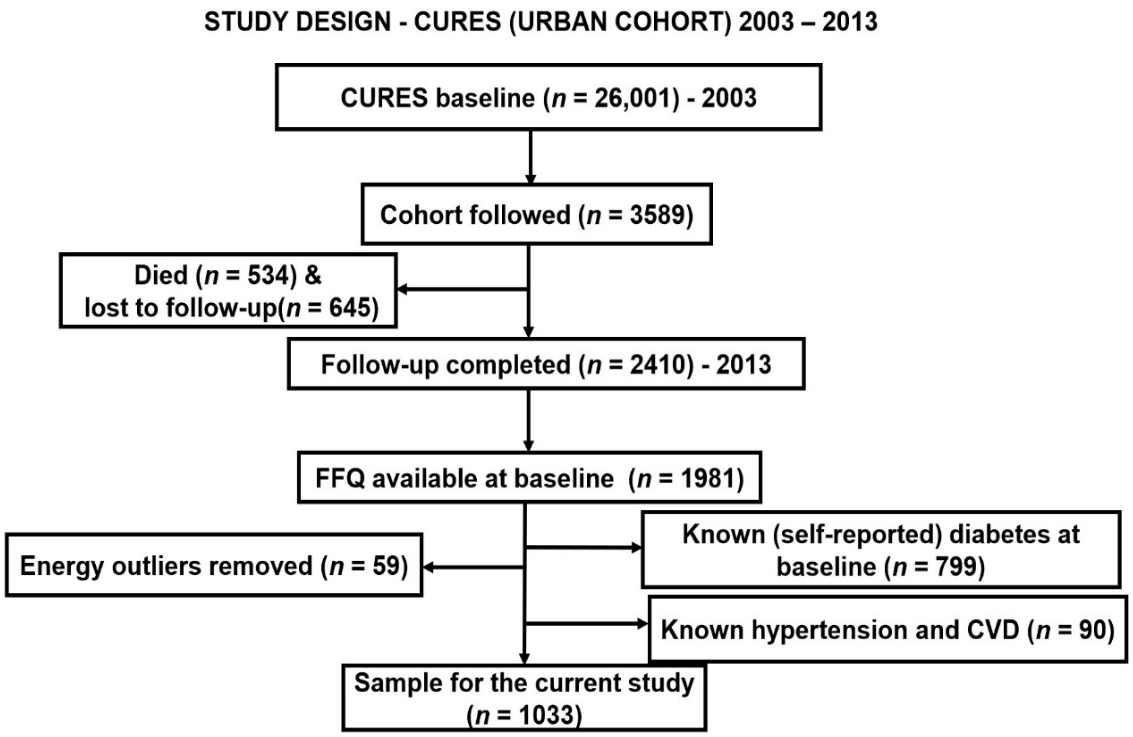

Supplementary Figure S1. A flow chart showing the selection of participants from the Chennai Urban Rural Epidemiological Study (CURES)

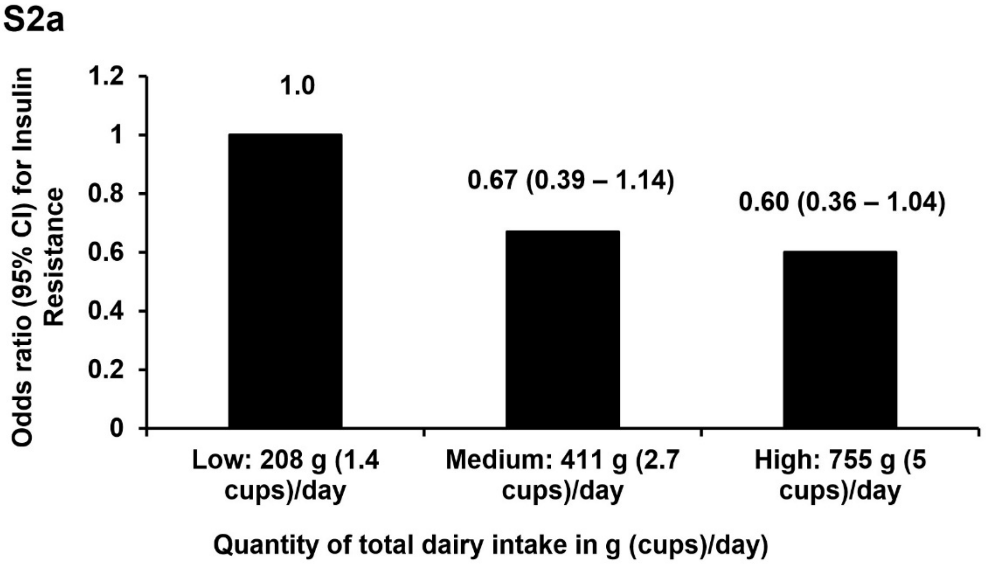

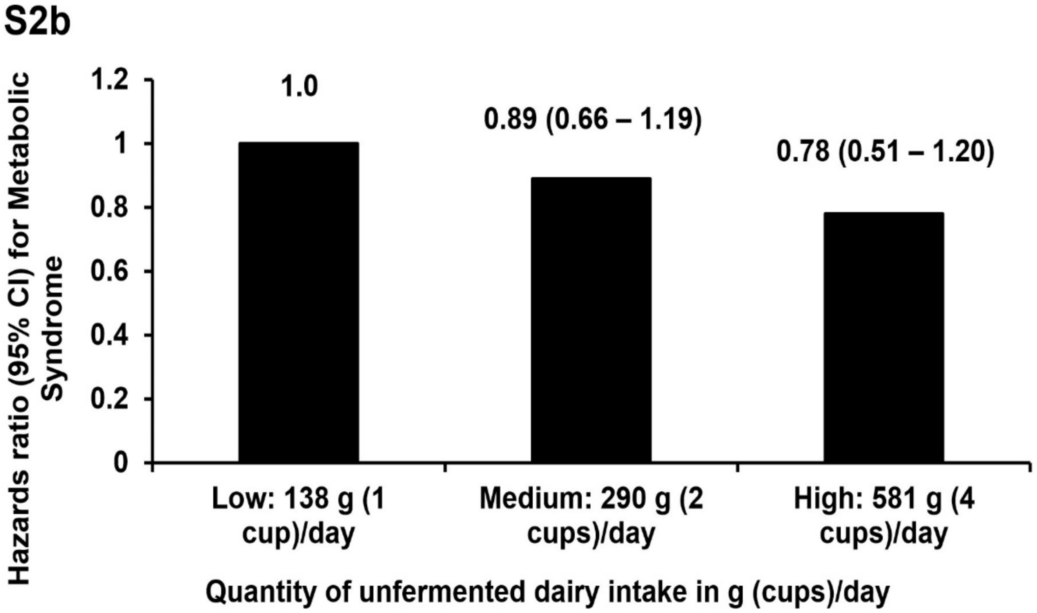

Supplementary Figure S2. (a) The association of total dairy intake with insulin resistance in the Chennai urban adults. Model adjusted for age (years), sex, income, weight (kg), alcohol, energy (kcal), added sugar(g), total fat(g), saturated fatty acid (g), tea and coffee intake.
(b) Unfermented dairy intake and its association with Metabolic Syndrome. Data presented as median. Adjusted variables are age (years), sex, BMI, income, smoking, alcohol, major cooking oil, total poly unsaturated fatty acids (PUFA) (g), added sugar (g), physical activity level, total energy (kcal), tea and coffee intake.
